# Aging and regional productivity growth in Germany

**DOI:** 10.1007/s10037-023-00188-3

**Published:** 2023-05-26

**Authors:** Eckhardt Bode, Dirk Dohse, Ulrich Stolzenburg

**Affiliations:** Kiel Institute for the World Economy, 24100 Kiel, Germany

**Keywords:** Workforce aging, Population aging, Productivity growth, Regional analysis, Germany, E24, J11, J24, J26, R11

## Abstract

We investigate the effects of aging on regional productivity growth, the mechanisms and the strength of which are not well-understood. We focus on two different manifestations of population aging—workforce aging and an increasing share of retirees—and investigate channels through which aging may impact on regional productivity growth for a panel of German counties 2000–2019. We find that workforce aging is more negatively associated with productivity growth in urban than in nonurban regions. A likely reason is that aging is detrimental to innovative and knowledge-intensive activities, which are heavily concentrated in cities. We also find a negative association between the share of the retired population and productivity growth in regions with a small household services sector. A likely reason is that older people’s disproportionate demand for local household services (including health care, recreation) requires a re-allocation of resources from more productive manufacturing or business services to less productive household services. Regions specialized more in highly productive industries have more to lose in this process.

## Abstract (de)

Die vorliegende Arbeit untersucht die Auswirkungen der Alterung auf das regionale Produktivitätswachstum, deren Mechanismen und Stärke noch nicht gut erforscht sind. Die Arbeit konzentriert sich auf zwei unterschiedliche Erscheinungsformen der Bevölkerungsalterung – die Alterung der Erwerbsbevölkerung und den zunehmenden Bevölkerungsanteil von Rentnern – und untersucht Kanäle, durch die sich die Alterung auf das regionale Produktivitätswachstum auswirkt. Die Analysen für ein Panel deutscher Landkreise 2000–2019 zeigen, dass zunehmende Alterung der Erwerbsbevölkerung in städtischen Regionen mit einem stärker sinkenden Produktivitätswachstum einhergeht als in nicht-städtischen Regionen. Ein Grund hierfür dürfte sein, dass die Alterung innovative und wissensintensive Tätigkeiten beeinträchtigt, die stark in Städten konzentriert sind. Die Analysen zeigen auch einen negativen Zusammenhang zwischen dem Anteil der Rentner und dem Produktivitätswachstum in Regionen mit einem kleinen Sektor der Haushaltsdienstleistungen. Ein Grund hierfür dürfte sein, dass die überdurchschnittliche Nachfrage älterer Menschen nach lokalen Haushaltsdienstleistungen (darunter Gesundheitsfürsorge und Erholung) einen Strukturwandel von produktiveren Sektoren wie Industrie oder Unternehmensdienstleistungen hin zu weniger produktiven Haushaltsdienstleistungen bewirkt. Regionen, die stärker auf hochproduktive Sektoren spezialisiert sind, haben in diesem Strukturwandel mehr zu verlieren.

## Introduction

Like the majority of OECD countries, Germany, Europe’s largest economy, is facing massive demographic challenges. Due to low birth rates among the native population that have only partly been compensated by immigration, the average age of the population increased continuously from about 41 years in the early 2000s to 44 years—interrupted only temporarily by the inflow of refugees in the mid-2010s from the Middle East and most recently from the Ukraine. The low birth rates have also continuously reduced the entry of young people into the labor market. While the average age of the workforce has increased by about 1.5 years since 2000, an acceleration is to be expected in the near future when the baby boomers reach their retirement age. According to the most recent population projection by the German Federal Statistical Office, the average age of the working-age population will continue to increase, the number of working age population will decrease (by between 1.6 and 4.8 million during the next 15 years, depending on migration). The number of retirees will increase by roughly 4 million to more than 20 million until the mid-2030s (Destatis 2022), by contrast.

**Fig. 1:**
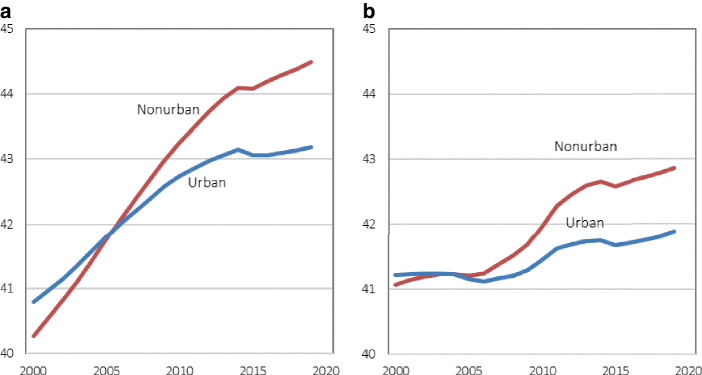
Average age of total and working age population in Germany by type of county. **a** Total population. **b** Working-age population (18–64 years). Note: Urban counties (114) and nonurban counties (287) as classified by the German Federal Institute for Research on Building, Urban Affairs and Spatial Development (BBSR, variable “Siedlungsstruktureller Regionstyp”, August 2018)

Moreover, aging has a distinct regional dimension, and the productivity effects of aging are likely to differ across regions. In Germany, there are stark regional differences in the age distributions and their changes over time. The average age of the population is much higher in East Germany than in West Germany. There are also considerable differences in the age compositions between urban and nonurban counties in Germany (Fig. 1a). While the average age of the population has increased quite substantially in both urban and nonurban counties, nonurban counties have been aging faster, which can be explained by the fact that aging in urban counties was partially offset by higher immigration. A similar difference is observed for the working age population (Fig. 1b). Its average age increased by almost two years in nonurban counties, compared to only 0.7 years in urban counties between 2000 and 2019. There are, however, large variations within the two groups of counties, ranging from a decrease of average age by 1.1 years to an increase by 5.4 years among the nonurban counties, and from a decrease of 1.8 years to an increase of 4.1 years among the urban counties.

In the present paper, we investigate the effects of aging on regional productivity growth, the mechanisms and the strength of which are not well-understood. We focus on two different manifestations of population aging, which are workforce aging and an increasing share of the non-working older population, and investigate the channels through which aging may impact on regional productivity growth for a panel of German counties 2000–2019. These channels are innovation and human capital, which relate to workforce aging, and sectoral changes toward household services, which relate to the increasing share of the retired population. We find that workforce aging is more negatively associated with productivity growth in urban than in nonurban regions. A likely reason for the higher sensitivity in urban regions is that aging is detrimental to innovative and knowledge-intensive activities, which are heavily concentrated in cities. We also find a negative association between the share of the retired population (aged 65+) and productivity growth in regions with a small household services sector. A likely reason is that older people’s disproportionate demand for local household services (including health care, recreation) requires a re-allocation of resources from more productive manufacturing or business services to less productive household services. Regions specialized more in highly productive industries have more to lose in this process.

The paper is structured as follows. Section 2 reviews the literature, summarizing its empirical results and discussing possible channels through which population aging may affect aggregate regional labor productivity growth. Section 3 develops the hypotheses and outlines the empirical strategy of the panel regressions for German counties. Section 4 presents the empirical results, and Section 5 summarizes the main findings and provides conclusions for policy.

## Aging and regional productivity growth: a brief review of the literature

Systematic empirical analyses of the consequences of aging for regional productivity growth are scarce.^1^ Most studies find a negative association between regional productivity or per-capita income growth and population aging. Brunow and Hirte (2006) regress per-capita income growth on the age composition of the population in a cross section of NUTS 2 regions in the EU 1995–2000. They find that per-capita income grows slower in regions with a higher share of the population aged 60–74, and faster in those with a higher share of the cohort aged 45–59, ceteris paribus. Their estimates are not directly informative about the effect of aging on labor productivity growth, though. Their estimated negative effect may be partly driven by lower labor market participation of the older cohort, which reduces GDP mechanically. Maestas et al. (2023) investigate this issue. Measuring aging in a panel of US states 1980–2010 by the share of the older population (aged 60+) in the total population aged 20+, they show that about one third of the negative effect of aging on per-capita income growth can be attributed to decreasing participation while only the remaining about two thirds can be attributed to decreasing productivity growth. They also show that aging tends to depress labor productivity in all age cohorts, which suggests spillovers or complementarities across age cohorts. Gabriele et al. (2018) also report a significantly negative elasticity of labor productivity with respect to the share of the older population (aged 55+) for a panel of Italian provinces 2009–2013 but this elasticity turns insignificant after instrumentation. In light of their short time period of only four years, which additionally were dominated by the global financial and the Euro crisis, it is not clear, however, to what extent these results are informative of the economic effects of aging, a gradual process that usually evolves slowly over long periods of time.

Brunow and Hirte (2009) is one of the few studies that explicitly study workforce aging at the regional level. They focus on aging of high-skilled workers, and use microdata to identify their distribution across age cohorts within German labor market areas. They find an inversely U‑shaped relationship across age cohorts when human capital is measured in terms of formal education (university degree) but a negative relationship when human capital is measured in terms of the skill requirements of the workers’ actual occupations.

Most closely related to the present paper is Daniele et al. (2019), who differentiate, like the present study, between urban and nonurban regions and investigate the growth effects of population aging from a reallocation of resources to less productive sectors. For a cross section of OECD regions 2001–2014, they find, like the present paper, that the negative effects of aging on productivity growth materialize primarily in urban regions. Unlike the present paper, they do not find significant growth effects of the sectoral reallocation toward less productive sectors. They exclude Germany from this analysis, however.

The literature emphasizes several channels through which population aging may impact on aggregate productivity growth at the level of regions.^2^ Workforce aging has been argued to work through mainly two channels. One of these channels is human capital of the workforce, which may establish a positive or negative relationship between aging of the workforce and aggregate labor productivity growth. In general, the productivity of individual workers increases over time through the accumulation of work experience and formal or informal training. This implies that, ceteris paribus, an older workforce tends to be more productive on aggregate than a younger workforce because it is more experienced. From the perspective of an individual region, aggregate productivity may consequently increase over time in spite of gradual aging of the workforce. However, the growth dynamics, i.e., the annual rate of aggregate productivity growth, will decrease mechanically in the course of aging, ceteris paribus. The reason for this mechanical decrease is that the relatively large share of more productive older workers in the total workforce who retire increases over time.

A second channel is innovativeness and entrepreneurial ambition of the workforce. The idea that young people are particularly capable of producing big ideas is common and longstanding (Planck 1949; Jones et al. 2014). A variety of studies^3^ find an inversely U‑shaped relationship between aging and innovation, start-up rates or firm dynamics while Piontek and Wyrwich (2017) report a negative effect of aging on entrepreneurial activities at universities in Germany.

Most of the literature suggests that the optimal age of innovators is around or slightly below 40 years. The pioneering work by Lehman (1953) finds that productivity peaks between ages 30 and 40. Jones (2010) finds that the average age at which great minds produce their greatest insights has increased over the last century to slightly below 40 in the majority of disciplines. According to Parker (2004), the golden age of entrepreneurship also occurs at the age of about 40. Azoulay et al. (2020) report that the mean founder age of high-growth enterprises in the US is somewhat higher (45 years), however.

In addition to affecting productivity growth through the generation of new ideas, aging may affect productivity growth through the adoption (or imitation) of these new ideas. Adoption of innovation by incumbent firms and workers depends on the willingness of employees and the management to adopt new technologies and new forms of work organization. Microeconomic evidence by Weinberg (2004) suggests that young, highly-qualified employees are better able to adapt to new technologies. While technology adoption complements experience among high school graduates, it complements youth among highly qualified employees (college and university graduates).^4^^,^^5^

These two channels associated with workforce aging can be expected to have a distinct regional dimension. Since human capital, innovation and high-growth entrepreneurship are highly concentrated in cities, the effects of workforce aging may impact stronger on urban than on rural areas. Within the last 30 years a rich empirical literature has developed showing that innovation has a pronounced tendency to cluster in urban regions.^6^ The high density of economic activity in cities facilitates the deliberate or unintended circulation of knowledge between economic actors, and the concepts of co-location and physical proximity are key to understanding the dynamics of the innovation process (Feldman and Kogler 2010). Innovation has been shown to be geographically more concentrated than production (Audretsch and Feldman 1996), and findings by Acs et al. (1994) suggest that new product innovations are even more geographically concentrated than patents. A key mechanism at work here is the increased importance of regionally embedded tacit knowledge (Maskell and Malmberg 1999), which is ‘sticky’ and very costly to transfer from place to place (Von Hippel 1994). Knowledge spillovers are localized and cities abundant in knowledge resources have been shown to provide a particularly fertile soil for the growth of young, technology-oriented firms (Audretsch and Dohse 2007). Moreover, urban diversity fosters the cross-fertilization of ideas which, in turn, enhances innovation (Jacobs 1969; Niebuhr 2010) and entrepreneurship (Audretsch et al. 2010). An important implication is that location has an impact on individual productivity, i.e., individuals with a given set of characteristics have different productivity levels depending on their location (Rigby and Essletzbichler 2002; De la Roca and Puga 2017). Doubling the size of a city causes productivity to increase by about 4% (Ahlfeldt and Pietrostefani 2019) and to increase patent output disproportionately (Bettencourt et al. 2007). The substantial advantages of cities in producing innovation and high-growth entrepreneurship and their specialization in these areas suggest that the effects of aging via the innovation and entrepreneurship channel impact stronger on urban areas. This is why we do not restrict our analysis of the effects of aging on productivity growth to the country level, but additionally differentiate between urban and nonurban regions in the empirical part of the paper.

An increasing share of retired population, the second manifestation of population aging, can affect labor productivity growth indirectly by older people’s specific consumption and savings behavior. The life cycle hypothesis (Modigliani and Brumberg 1954) suggests that individuals smooth their consumption over the life cycle, consuming more than they earn at young and old ages and funding these excess expenditures by accumulating wealth at middle ages while they are working. Hence, populations with increasing older population tend to have decreasing savings rates (Börsch-Supan et al. 2006), which could theoretically lead to a decreasing regional capital supply and depress the marginal product of labor (e.g., Choudhry et al. 2016). However, given the high interregional capital mobility, this is unlikely to happen in countries like Germany. Moreover, potential effects from lower savings rates of the elderly might be offset by increasing life expectancy that tends to increase savings over the life cycle and thus capital supply (e.g., Daniele et al. 2019).

Older people’s consumption behavior, our third channel, may be more relevant. Older people arguably tend to demand more local services, including health care and recreational services. To meet this increasing demand, aging regions may experience a disproportionate sectoral shift towards the household services sector. This sectoral shift likely depresses labor productivity growth because services industries are more labor intensive and less productive than other sectors like manufacturing.^7^ Hence, the aging-induced sectoral shift towards household services may depress growth particularly strongly in highly productive manufacturing regions whereas regions with an already high share of (relatively less productive) services are likely to lose less from this transition.

## Research questions and empirical framework

### Research questions

This section formulates test hypotheses to guide our empirical analysis, which focuses on the three main channels just discussed, the human-capital and innovation channels (1 and 2), which we address jointly because we cannot disentangle them empirically, and the consumption channel (3). We begin by examining the relationship between workforce aging and labor productivity growth on aggregate across all regions and then investigate whether the effect of workforce aging in urban regions differs systematically from the effect in nonurban regions. In the third step we investigate the consumption channel.

As discussed in the preceding section, the net effect of workforce aging on labor productivity is theoretically ambiguous. It depends on the trade-off between the positive effect of increasing aggregate human capital (work experience) and the negative effect of increasing losses of human capital through retirement. It additionally depends on the association between workers’ age and their innovativeness, which has been found to peak at ages somewhat below 40 years in most studies. In addition to this, the adaptive capacity may decrease with aging. Since the average age of the workforce has already well exceeded 40 years in the vast majority of German regions, we expect that increasing retirement and decreasing innovativeness dominate human capital gains from aging in Germany, which leads us to our first test hypothesis.

#### H1

Workforce aging in Germany is negatively associated with labor productivity growth.

The second hypothesis addresses the possible differences between urban and nonurban regions. As a larger share of value-added in urban regions depends on the creation and adoption of innovation, productivity growth can be expected to be more sensitive to workforce aging in urban than in nonurban regions. While the average age of the workforce is somewhat lower in urban than in nonurban regions, it has already moved beyond the peak age for both the generation of innovations (which is somewhat below 40 years) and the adoption of new (digital) technologies (which is likely even lower). We thus hypothesize:

#### H2

Productivity growth is more sensitive to workforce aging in urban than in nonurban regions.

Population aging does not only affect the workforce but also implies an increasing share of older (presumably non-working) people. As discussed in the literature review, this group can affect labor productivity growth indirectly through its specific consumption behavior. An increasing share of the retired population (aged 65+) can increase the share of the household services sector, in particular health care and other social services as well as recreational services, at the expense of other sectors such as manufacturing or business services (channel 3). As household services tend to have a lower average productivity than manufacturing and business services, the effect of this structural shift on productivity growth should be negative. This negative effect should be larger in regions that have a lower share of household services (and a higher share of highly productive manufacturing or business services) as these regions have a higher aggregate labor productivity and have thus more to lose from a structural shift towards low-productivity household services.

#### H3

A higher share of the older non-working age population is negatively associated with labor productivity growth. This negative effect is stronger in regions with higher manufacturing or business services shares and weaker in regions with a higher household services share.

### Empirical framework

To test the three hypotheses H1–H3 empirically, we regress long-term productivity growth on indicators of the respective manifestations of population aging for a panel of the 401 German counties (NUTS level 3) during the period 2000–2019. Our baseline model, which focuses on the growth effects of workforce aging (H1), can be written as

1 $$\hat{y}_{rt}=\alpha _{0}+\beta \ln \left(\mathrm{age}_{rt}\right)+\boldsymbol{X}_{\boldsymbol{rt}}\boldsymbol{\gamma }+\iota _{r}+\iota _{t}+\varepsilon _{rt}$$

where *r* indexes counties and *t* years (2000–2019, five-year intervals). $\hat{y}_{rt}=\left(\ln y_{rt+\tau }-\ln y_{rt}\right)$/*τ* denotes productivity growth, defined as the average annual growth rate of GDP per worker from year *t* to $t+\tau$. The variable *age*_*rt*_ is average age of the workforce in the initial year *t*, our main indicator of workforce aging; **X**_**rt**_ is a vector of control variables; *ι*_*r*_ and *ι*_*t*_ are full sets of county and time fixed effects; and *ε*_*rt*_ is the error term. We cluster standard errors at the level of NUTS 2 regions in order to account for possible serial autocorrelation over time as well as spatial autocorrelation across the counties within their NUTS 2 region.^8^

In contrast to several of the earlier studies reviewed in Section 2, we explicitly do not root our empirical model in a specific theoretical model of economic growth. A neoclassical model of growth and convergence, as used by Brunow and Hirte (2006), among others, imposes strong restrictions on functional form and covariates that may mask the empirical relationship between aging and productivity growth. One particularly restrictive feature of these theoretical models is that constant returns to scale and the lack of interregional exchange of factors and products establish some mechanics of income convergence for each region. Exogenously imposing such a mechanical convergence process on a model that aims at studying another fairly mechanical process may be problematic. As Feyrer (2007, p. 101) put it, “Since the age structure of the population will vary over time in a very structured way (the size of the group aged 30–35 today is roughly the same as the group aged 25–30 five years ago), it is not clear how to interpret the results of a regression that includes lagged dependent variables.”

The time period we focus on, 2000–2019, excludes the 1990s that were characterized by strong economic turbulences following the fall of the iron curtain and the German reunification. By 2000, the restructuring of the East German economy was not yet fully completed, the infrastructure deficits in East Germany were not yet completely eliminated, and there was still some migration from East to West Germany. However, the economic situation had stabilized considerably. The period 2000–2019 also excludes the time of the Covid-19 pandemic, which started in early 2020 and saw massive public interventions to avoid bankruptcies and mass layoffs in spite of a significant economic downturn. Rather than estimating the growth effects of aging over the entire time span of 20 years in a cross-section setting, we divide this period into four five-years’ time intervals, 2000–2005, 2005–2010, 2010–2015 and 2015–2019 (four years). These time intervals are long enough to smooth temporary local shocks and still grant us a decent panel dimension of four observations. Importantly, the panel setting allows us to control for unobserved heterogeneity across regions and over time by fixed effects. The county fixed effects, *ι*_*r*_ in Eq. 1, ensure that the growth effects of aging are identified only from the variation within counties over time. The time period fixed effects, *ι*_*t*_, ensure that our parameters are not affected by national variations over time in, among others, business cycles, inflation or technological progress that affect productivity growth in all counties.

In the cross-section dimension, we observe data from all 401 German counties (Landkreise and kreisfreie Städte) in their current territorial delimitation. The panel is unbalanced, though, because we had to exclude six counties from the state of Sachsen-Anhalt until 2010 and two from Mecklenburg-Vorpommern until 2015 due to changes of the territorial delimitation that cut through former counties.

As to the dependent variable, we focus on labor productivity rather than per-capita income growth to abstract from the pure level effect induced by the fact that aging reduces GDP mechanically by reducing the population that contributes to producing it (Maestas et al. 2023). And we focus on productivity growth rather than the productivity level to abstract from the fact that the productivity level differs between regions and may increase just because aging skews the distribution of the workforce towards older, more experienced workers.^9^ In combination with county fixed effects this also eliminates the effects of unobserved drivers of productivity growth that may be correlated with aging.

Our variable of main interest is workforce aging, which we measure by the (logged) average age of the workforce, ln(age_*rt*_). Its parameter, $\beta =\partial \hat{y}_{rt}/\partial \ln \left(\mathrm{age}_{rt}\right)$, reflects the dynamic effect of workforce aging on growth in terms of a marginal increase of average age on productivity growth.^10^ Since data on the age composition of the workforce is not available for a sufficiently long period of time, we approximate it by the age composition of the working-age population.^11^ As a robustness check, we alternatively use the share of older workers aged 50–64 years in the working age population as a measure of aging. The results, reported in Table 4 in the Appendix, are qualitatively very similar.

A variety of control variables, **X**_**rt**_ in Eq. 1, are included to avoid omitted variables biases of our parameter of main interest. We control for the growth rates and initial-year levels of the counties’ population sizes, labor participation rates and sectoral value-added shares.^12^ The (logged) initial-year level and the growth rate of the population control, among others, for agglomeration economies as well as for migration and fertility. The (logged) initial-year level and the growth rate of the participation rate, which is measured as the population share of the workforce, control for changes in labor market participation, e.g., through unemployment, that may mitigate or aggravate the productivity effects of aging. The (logged) initial-year levels and the growth rates of the shares of the manufacturing sector, business services and household services in value added control for the productivity effects of structural changes.^13^

To test hypotheses H2 and H3, we successively modify model (1) by estimating the effects of aging separately for 114 urban and 287 nonurban counties (H2),^14^ and by adding the (logged) share of retired workers aged 65+ as well as its interaction with the sectoral composition of the regional economy (value added shares of manufacturing, business and household services) as explanatory variables (H3).^15^

The Appendix reports data sources and descriptive statistics in Tables 2 and 3. It additionally reports a battery of further regressions in Tables 4 and 5, which show that (i) measuring workforce aging in terms of the share of older workers (aged 50–64 years) in the workforce rather than by average age, or (ii) controlling additionally for spatial lags^16^ of all regressors do not affect the inferences drawn below to a notable extent.

## Regression results

Table 1 reports our main results of the tests of hypotheses H1–H3. The first column reports the results for the baseline model (1), which addresses the association between workforce aging and productivity growth on average across all regions (H1). The parameter of average age of the workforce is negative (−0.047), which suggests that workforce aging tends to depress regional productivity growth. It is measured rather imprecisely, though (prob-value: 0.14), which is due to heterogeneity between urban and nonurban counties, as will become clear soon. The point estimate implies that an increase of the average age of the workforce by one year is associated with a roughly 0.1 percentage points lower annual productivity growth.^17^

**Table 1:** Regression results: Aging and productivity growth in German counties 2000–2019

	(1)	(2)	(3)	(4)
Average age working-age population	−0.047 (0.031)	–	–	–
Average age working-age population urban	–	−0.087** (0.035)	−0.084** (0.033)	−0.064* (0.033)
Average age working-age population nonurban	–	−0.042 (0.032)	−0.039 (0.029)	−0.027 (0.029)
Share population aged 65+	–	–	−0.001 (0.006)	0.027 (0.034)
Share population aged 65 + x share manufacturing	–	–	–	0.001 (0.013)
Share population aged 65 + x share business services	–	–	–	−0.001 (0.007)
Share population aged 65 + x share household services	–	–	–	0.022** (0.010)
Population growth	0.616*** (0.108)	0.609*** (0.108)	0.612*** (0.108)	0.603*** (0.102)
Growth participation rate	0.479*** (0.048)	0.481*** (0.049)	0.482*** (0.049)	0.507*** (0.051)
Growth manufacturing value added share	−0.017 (0.014)	−0.017 (0.014)	−0.017 (0.014)	−0.018 (0.013)
Growth business services value added share	−0.297*** (0.035)	−0.293*** (0.035)	−0.292*** (0.035)	−0.290*** (0.036)
Growth household services value added share	−0.540*** (0.034)	−0.539*** (0.034)	−0.539*** (0.034)	−0.537*** (0.034)
Population size	0.005 (0.010)	0.005 (0.010)	0.005 (0.010)	0.014 (0.010)
Participation rate	0.004 (0.008)	0.005 (0.008)	0.005 (0.008)	0.013 (0.008)
Manufacturing value added share	−0.003 (0.003)	−0.003 (0.003)	−0.003 (0.003)	−0.005 (0.014)
Business services value added share	−0.009 (0.007)	−0.009 (0.007)	−0.009 (0.007)	−0.009 (0.023)
Household services value added share	−0.002 (0.006)	−0.002 (0.006)	−0.002 (0.006)	0.035* (0.019)
Constant	0.126 (0.215)	0.156 (0.210)	0.147 (0.200)	0.036 (0.197)
R‑squared	0.769	0.770	0.770	0.772

Fixed effects panel regressions (within estimators), dependent variable: average annual growth rate of GDP per worker, periods: 2000–2005, 2005–2010, 2010–2015, 2015–2019; unbalanced panel: number of observations: 1586; number of counties: 401. All explanatory level variables are in logs, all growth rates are in log-differences. All regressions include full sets of county and time period fixed effects. Standard errors (in parentheses) are clustered at the level of NUTS 2 regions

Significance levels: *: 10%; **: 5%; ***: 1%

The parameters of the control variables suggest plausibly that, conditional on aging, productivity growth increases with increasing population size, possibly due to agglomeration economies, and increasing labor market participation rates, possibly due to some efficiency gains. They also suggest that aggregate productivity growth is sensitive to structural changes. Compared to that of the reference sectors (agriculture and construction), disproportionate expansions of business services or household services in terms of the growth of their value-added shares depress aggregate productivity growth significantly (−0.3, resp. −0.54) because productivity growth is slower in these services industries. A disproportionate expansion of the manufacturing sector does not affect aggregate productivity growth to a notable extent, by contrast (parameter: −0.017).

Column (2) of Table 1 addresses our second hypothesis, which holds that productivity growth is more sensitive to workforce aging in the 114 urban regions than in the 287 nonurban regions. The results suggest that the negative effect of workforce aging on productivity growth is larger in urban (−0.087) than in nonurban counties (−0.042). The parameter for the urban counties is highly significant (prob-value: 0.015) while that for the nonurban counties is measured rather imprecisely (prob-value: 0.19).^18^ Evaluated at the subsample averages in 2000 (41 years), the point estimate for urban counties suggests that an increase of average age by one year is associated with a drop of annual productivity growth by 0.21 percentage points.^19^ As discussed in Section 2, a likely reason for this higher sensitivity of growth in urban regions is that urban regions, due to their specialization in innovative activity, suffer more from the age-related decline of workers’ innovativeness and adaptability to technological and organizational changes. Due to their specialization in human capital, they may additionally suffer more from losses of experienced workers through retirement.

Finally, columns (3) and (4) address H3, which states that a growing share of the older population (aged 65+) leads to increased consumption of household services, thereby dampening productivity growth. To start with, we add the (logged) share of the population aged 65+ in column (3) to assess the direct effect of retirees on productivity growth, and interact this share with our indicators of the sectoral composition of the regional economy (value added shares of manufacturing, business services and household services) in column (4) to assess complementarities between retirees and the industry composition.

**Fig. 2:**
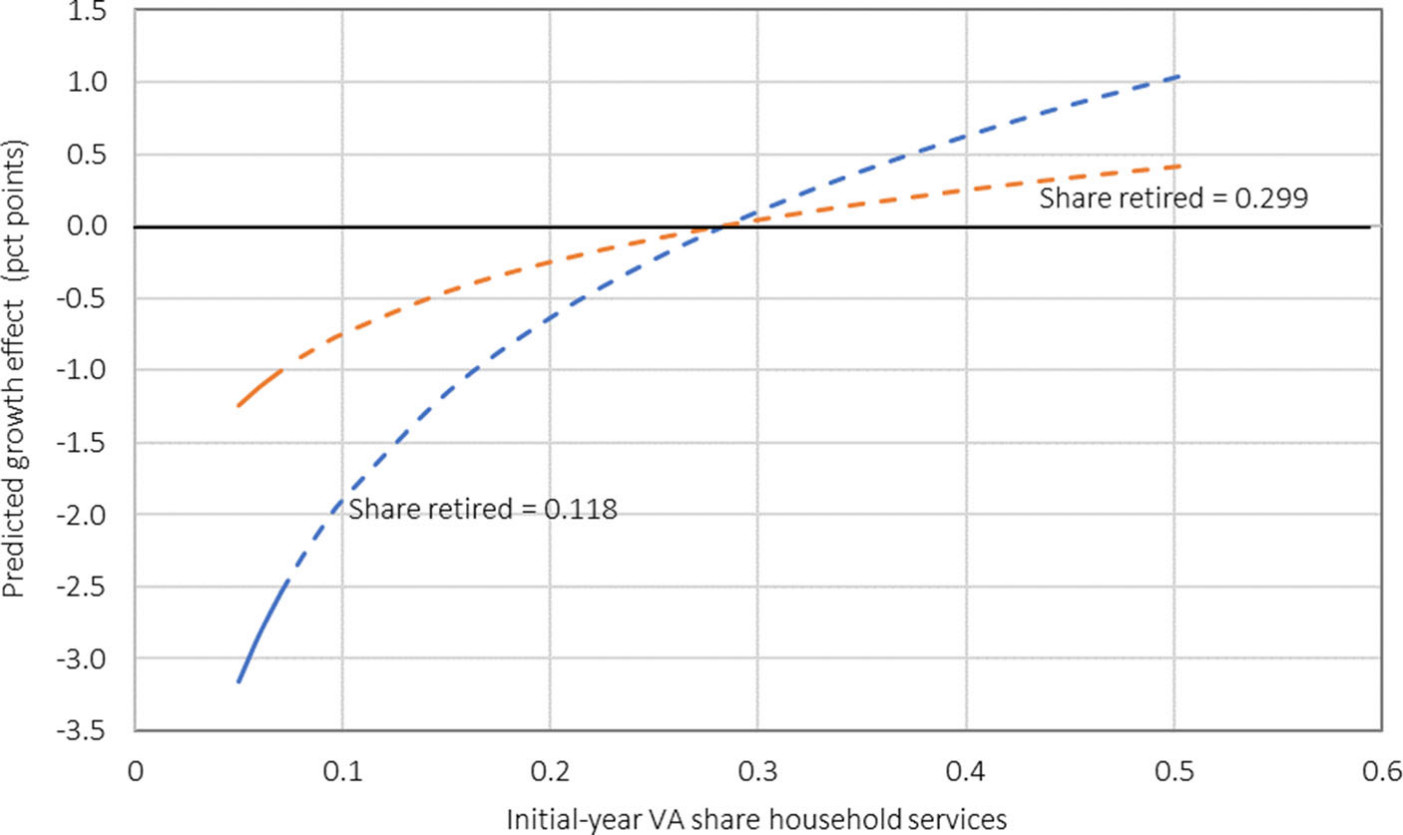
Predicted growth effect of a 10 percentage points increase of the share of the retired population. Notes: The predicted growth effect accounts for the direct and the indirect effect through household services of a 10 percentage points increase of the share of the retired population for different sizes of the household services sector and the lowest (0.118, *blue curve*) and highest population shares of retirees (0.299, *red curve*) observed in the sample. *Solid parts of the curves:* significant at the 5% level; *dashed parts of the curves:* not significant at the 5% level

The results indicate, first, that a higher share of retirees is not associated directly with productivity growth. The parameter of the share of the population aged 65+ is clearly insignificant in both regressions. Second, however, we estimate a significant interaction between retirees and household services in their growth effect (0.022). Since the magnitude of the growth effect of this complementarity is not easily inferred from the point estimates because it depends on both the share of retirees and the size of the household services sector, Fig. 2 shows the predicted effect of an increase of the share of retirees by 10 percentage points on productivity growth implied by these estimates.^20^ The horizontal axis depicts the range of household services shares observed in our sample (5.7–51.4%; see Table 2 in the Appendix), while the two lines show the predicted effects for the smallest (11.8%, blue line) and the largest population share of retirees in the sample (29.9%, red line). The solid parts of the curves indicate that the predictions are significant at the 5% level (dashed parts: prob > 0.05). The figure shows that the growth effects are estimated to be negative and significant for counties with an initially small household services sector but positive (though insignificant) for those with a large household services sector. It also shows that the growth effects become weaker with increasing population share of retirees.

Thus, productivity growth is, according to our estimates, particularly sensitive to aging in terms of an increasing share of the retired population in those regions that both have a small retired population and whose industry composition is rather poorly equipped to accommodating the needs of the older population in terms of household services. One plausible reason for the higher age-sensitivity of growth in these regions is that aging requires a stronger structural change that reallocates resources from more productive manufacturing or business services to less productive household services. This growth-inhibiting reallocation of resources appears to be less of a problem for regions that are already well endowed with household services, by contrast. An already large household services sector may be able to meet the growing demand of an aging population more easily, and may even realize economies of scale or scope that foster rather than inhibit productivity growth.

Notice that the productivity growth effect we attribute to structural change toward household services induced by population aging is actually very similar to the productivity growth effects of structural change toward household services induced by other forces. In our estimations, the latter are reflected by the control variables, which capture the growth effects of structural changes irrespective of the forces that drive them. The parameters of the control variables suggest that, ceteris paribus and on average across all counties, a faster structural shift to household services (from the reference sectors, agriculture and construction) is associated with lower aggregate productivity growth (parameter −0.537 in column 4 of Table 1), and that this growth penalty is somewhat lower in counties with a higher (initial-year) share of household services (parameter 0.035), resp. somewhat higher in counties with a lower share of household services. The growth effect of structural change toward household services driven by any economic forces is thus similar to the one we attribute to population aging.

Importantly, while the association between workforce aging and productivity growth in urban counties becomes somewhat weaker when the growth effect of the population aged 65+ is accounted for, it is still negative (−0.064 in regression 4) and significant (prob-value: 0.056). Even though these two manifestations of population aging are fairly high correlated with each other,^21^ our results indicate that they affect regional productivity growth through different channels in Germany: Population aging in terms of workforce aging reduces growth presumably by reducing the innovativeness and adaptive capacity of the workforce while population aging in terms of a growing size of the cohort of retirees reduces growth presumably by retirees’ disproportionate consumption of less productive household services.

Our main results remain largely unchanged when we measure workforce aging by the share of older workers (aged 50–64 years) rather than by average age (Table 4 in the Appendix).

## Conclusions and implications for policy

Germany, Europe’s largest economy, is a showcase of an aging society. Yet, the economic consequences of aging are not well understood, although they are likely to have important implications for innovativeness, growth and political stability at the national and regional levels. In this paper, we contribute to a better understanding of the effects of population aging on regional productivity growth. We discuss several channels through which two manifestations of population aging—workforce aging and a growing share of the non-working older population—might affect regional productivity growth and assess the empirical relevance of these channels using a panel of German counties 2000–2019.

We find a negative association between workforce aging and productivity growth at the regional level. The type of region plays an important role in this context. Productivity growth is more sensitive to workforce aging in urban than in nonurban regions. One plausible reason for this higher sensitivity is that workforce aging weakens innovative and knowledge-intensive activities, which are highly concentrated in cities. Although the average age of the workforce is slightly lower in urban than in nonurban regions, it is already above the age considered optimal in the literature for generating and adopting innovation.

We also find a negative association between the share of the older population (aged 65+) and regional labor productivity growth in regions with a small household services sector. One plausible channel for this association is the specific demand patterns of older people. To satisfy older people’s disproportionate demand for local household services, especially health care and recreational services, aging economies have to undergo an accelerated structural shift towards these services. Since the productivity level and productivity growth are lower in these services than in most other sectors, this structural shift slows down aggregate productivity growth. Regions with a still small household services sector have more to lose in this process because they have to reduce their specialization in more productive industries, notably manufacturing.

Taken together, these findings are good news and bad news in one. They are bad news as urban and manufacturing regions are the engines of innovation and the backbone of the German export industry. Factors that dampen productivity growth in these regions challenge the innovativeness and international competitiveness of the German export sector and the German business model as a whole. The good news is that urban regions are the economically strongest and (from the perspective of potential migrants) most attractive regions, which opens up strategic opportunities and gives policy makers some latitude to implement adequate policy measures.

A policy that counteracts workforce and population aging at its roots is fostering immigration of skilled young workers on the one hand and immigration of young families from countries with higher fertility rates on the other. The supply of skilled young immigrants from Europe is rather limited, however. Population and workforce aging also affect many of the potential European countries of origin. Firms and locations all over Europe compete for young, highly qualified people. The current German immigration system that does not clearly distinguish between humanitarian migration and labor migration appears rather inefficient from a labor market point of view. To become more successful in the global competition for talent, a targeted points-based immigration system like those in Canada or New Zealand might help. The German government has been taking steps in this direction in recent years but major administrative and bureaucratic barriers to immigration remain, including the requirement for recognition of immigrants’ vocational qualifications (e.g., Adunts et al. 2023). Moreover, new approaches like the ‘global skill partnership’ model (discussed in Clemens et al. 2019) that provides training to potential immigrants in their home countries before migration, along with non-migrants, might prove beneficial for both destination and origin countries.

Apart from migration policy, mobilizing untapped internal labor market resources is key to prevent shortages of qualified labor and slowdown of productivity (Bickenbach et al. 2022). This includes, among others, to improve the quality of the education system, to establish a culture of life-long learning, to upskill low-skilled workers, to increase female participation^22^, notably in STEM jobs, to better integrate forced migrants (asylum-seekers) into the labor market, and to tap the potential of older people. Which measures are most adequate depends on regional circumstances, and regional policymakers should be given sufficient latitude to adapt policies to their region’s specific needs and assets.

Still, there is need for further research that helps to better substantiate economic policies to strengthen economic growth and well-being in aging societies. The current paper has analyzed some of the most plausible channels through which population aging affects regional productivity growth, hypothesizing that workforce aging hampers growth by reducing workers’ ability to come up with, or adapt new ideas, and that population aging hampers growth by fostering structural change toward less productive household services. However, more theoretical and empirical work is warranted to identify the relative importance of these channels, and to identify opportunities to foster innovation and entrepreneurship in aging societies. In addition to this, additional plausible channels through with aging may affect productivity growth need to be explored in more detail. One of these channels is human capital formation among older workers. More extensive, targeted training of older workers in new technologies may counteract the negative growth effects of decreasing innovativeness in the course of workforce aging. Another channel is automation of production by robots and other digital technologies, which have the potential to increase productivity and allow human resources to be reallocated to other activities, including services for the elderly.

## Appendix

**Table 2:** Descriptive statistics for growth regressions: German counties 2000–2019

Variable	N	Mean	Std. dev	Min	Max
*Growth rates (avg. annual)*					
Labor productivity growth	1604	0.026	0.014	−0.032	0.134
Population growth	1604	0.000	0.006	−0.026	0.020
Growth participation rate	1604	0.005	0.009	−0.026	0.033
Growth manufacturing value added share	1604	−0.004	0.030	−0.185	0.385
Growth business services value added share	1604	0.000	0.015	−0.129	0.056
Growth household services value added share	1604	0.001	0.015	−0.145	0.078
*Initial-year levels (logs)*					
Average age of workforce (aged 18–64)	1586	3.731	0.027	3.633	3.830
Share older workers (aged 50–64) in workforce	1586	−1.147	0.128	−1.507	−0.739
Share population aged 65+	1586	−1.631	0.144	−2.133	−1.207
Population size	1604	11.972	0.647	10.438	15.067
Participation rate	1604	−0.735	0.261	−1.435	0.223
Manufacturing value added share	1604	−1.559	0.525	−4.809	−0.265
Business services value added share	1604	−0.873	0.199	−1.890	−0.241
Household service value added share	1604	−1.498	0.312	−2.865	−0.666
*Initial-year levels, un-logged*					
Average age of working-age population (18–64)	1586	41.752	1.128	37.809	46.076
Share older workers in working-age population	1586	0.320	0.042	0.222	0.478
Share population aged 65+	1586	0.198	0.028	0.118	0.299
Population size	1604	202,471	225,070	34,136	3,494,940
Participation rate	1604	0.497	0.146	0.238	1.249
Manufacturing value added share	1604	0.237	0.110	0.008	0.767
Business services value added share	1604	0.426	0.084	0.151	0.786
Household service value added share	1604	0.234	0.07	0.057	0.514

All Initial-year levels enter our regressions in logs

**Table 3:** Correlation matrix for growth regressions: German counties 2000–2019

	Variable	1	2	3	4	5	6	7	8	9	10	11	12	13
1	Labor productivity growth	1.00												
2	Average age of workforce (ln)	0.13	1.00											
3	Share older workers (aged 50–64) (ln)	0.20	0.93	1.00										
4	Share population aged 65+ (ln)	0.17	0.65	0.72	1.00									
5	Population growth	0.28	−0.23	−0.11	−0.25	1.00								
6	Growth participation rate	0.37	0.13	0.12	0.27	−0.18	1.00							
7	Growth manufacturing value added share	0.34	−0.01	−0.03	0.03	−0.10	0.11	1.00						
8	Growth business services value added share	−0.48	−0.15	−0.18	−0.28	0.06	−0.29	−0.48	1.00					
9	Growth household services value added share	−0.66	−0.03	0.00	−0.02	0.05	−0.16	−0.38	0.28	1.00				
10	Population size (ln)	−0.01	−0.12	−0.14	−0.19	0.16	0.01	−0.04	0.04	0.06	1.00			
11	Participation rate (ln)	0.06	−0.42	−0.23	0.05	0.33	−0.13	−0.02	−0.03	0.05	−0.09	1.00		
12	Manufacturing value added share (ln)	0.02	0.03	0.01	−0.07	−0.04	0.09	−0.08	0.10	−0.04	−0.06	−0.02	1.00	
13	Business services value added share (ln)	0.01	−0.12	−0.13	−0.10	0.29	0.00	−0.02	−0.14	0.11	0.31	0.03	−0.60	1.00
14	Household service value added share (ln)	−0.04	−0.02	0.06	0.25	−0.21	−0.10	0.04	−0.07	−0.07	−0.20	0.04	−0.61	0.09

**Table 4:** Additional regression results: Share older workers

	(1)	(2)	(3)	(4)
Share older workers (50–64 years)	−0.009 (0.007)	–	–	–
Share older workers urban	–	−0.013* (0.007)	−0.012* (0.007)	−0.011* (0.007)
Share older workers nonurban	–	−0.009 (0.007)	−0.008 (0.007)	−0.008 (0.006)
Share population aged 65+	–	–	−0.004 (0.007)	0.023 (0.035)
Share population 65 + x share manufacturing	–	–	–	−0.002 (0.013)
Share population 65 + x share business services	–	–	–	−0.002 (0.007)
Share population 65 + x share household services	–	–	–	0.022** (0.010)
Population growth	0.624*** (0.108)	0.617*** (0.109)	0.621*** (0.109)	0.599*** (0.103)
Growth participation rate	0.483*** (0.048)	0.484*** (0.048)	0.486*** (0.048)	0.512*** (0.051)
Growth manufacturing value added share	−0.016 (0.014)	−0.016 (0.014)	−0.016 (0.014)	−0.018 (0.013)
Growth business services value added share	−0.295*** (0.034)	−0.293*** (0.035)	−0.292*** (0.035)	−0.291*** (0.036)
Growth household services value added share	−0.538*** (0.034)	−0.537*** (0.034)	−0.538*** (0.034)	−0.536*** (0.034)
Population size	0.007 (0.010)	0.007 (0.009)	0.006 (0.010)	0.014 (0.010)
Participation rate	0.005 (0.008)	0.005 (0.008)	0.005 (0.008)	0.014* (0.008)
Manufacturing value added share	−0.003 (0.003)	−0.003 (0.003)	−0.003 (0.003)	−0.006 (0.014)
Business services value added share	−0.009 (0.007)	−0.008 (0.007)	−0.008 (0.007)	−0.014 (0.023)
Household services value added share	−0.002 (0.006)	−0.001 (0.006)	−0.001 (0.006)	0.036* (0.019)
Constant	−0.082 (0.108)	−0.082 (0.107)	−0.076 (0.109)	−0.122 (0.109)
R‑squared	0.769	0.769	0.769	0.772

Fixed effects panel regressions (within estimators), dependent variable: average annual growth rate of GDP per worker, periods: 2000–2005, 2005–2010, 2010–2015, 2015–2019; unbalanced panel: number of observations: 1586; number of counties: 401. All explanatory level variables are in logs, all growth rates are in log-differences. All regressions include full sets of county and time period fixed effects. Standard errors (in parentheses) are clustered at the level of NUTS 2 regions

Significance levels: *: 10%; **: 5%; ***: 1%

**Table 5:** Additional regression results: Spatially lagged regressors

	(1)	(2)
Average age working-age population	−0.045 (0.031)	–
Spatially lagged average age working-age population	0.003* (0.002)	0.003* (0.002)
Average age urban	–	−0.087** (0.034)
Average age nonurban	–	−0.038 (0.0317)
Population growth	0.777*** (0.111)	0.770*** (0.112)
Growth participation rate	0.520*** (0.053)	0.523*** (0.054)
Growth manufacturing value added share	−0.016 (0.014)	−0.016 (0.014)
Growth business services value added share	−0.290*** (0.036)	−0.286*** (0.036)
Growth household services value added share	−0.552*** (0.036)	−0.552*** (0.036)
Population size	0.011 (0.015)	0.010 (0.015)
Participation rate	0.003 (0.008)	0.003 (0.009)
Manufacturing value added share	−0.003 (0.003)	−0.003 (0.003)
Business services value added share	−0.007 (0.007)	−0.007 (0.007)
Household services value added share	−0.004 (0.006)	−0.004 (0.006)
Constant	0.09 (0.212)	0.111 (0.207)
R‑squared	0.775	0.775

Fixed effects panel regressions (within estimators), dependent variable: average annual growth rate of GDP per worker, periods: 2000–2005, 2005–2010, 2010–2015, 2015–2019; unbalanced panel: number of observations: 1586; number of counties: 401. All explanatory level variables are in logs, all growth rates are in log-differences. All regressions additionally include full sets of county and time period fixed effects as well as spatial lags of all control variables. The estimates for the spatial lags of the control variables are omitted from this table to save space but are available from the authors upon request. The spatial lags are calculated using a row-standardized binary first-order contiguity matrix (direct neighbors). Standard errors (in parentheses) are clustered at the level of NUTS 2 regions

Significance levels: *: 10%; **: 5%; ***: 1%

## Footnotes

1 See Gabriele et al. (2018) and Daniele et al. (2019) for a review of the early literature. Most studies at the country-level find an inverted U‑shaped association between aging and productivity growth (e.g., Lindh and Malmberg 1999; Feyrer 2007; Liu and Westelius 2016; Aiyar et al. 2016). Acemoglu and Restrepo (2018), who find a positive relationship, is an exception.

2 Focusing on labor productivity growth rather than per-capita income growth, we abstract from the mechanical effects of aging on GDP growth through labor market participation here.

3 See, among others, Bönte et al. (2009), Meyer (2011), Gregory and Patuelli (2015), Pfeifer and Wagner (2014), Liang et al. (2018), Azoulay et al. (2020).

4 Related to both the human capital and the innovation channel may be spillovers across age groups of the workforce. Maestas et al. (2023) show that population aging slows down productivity growth in all age groups. This may be due to what Liang et al. (2018), who find a similar correlation across age groups for start-up rates, label the “rank effect”. In older societies, a larger fraction of the positions that are particularly conducive to the accumulation of qualified work experience or entrepreneurial skills, are likely occupied by older workers. This reduces the opportunity of younger workers for enhancing their productivity or successfully starting their own ventures. This relative lack of opportunities at younger ages has a permanent effect on productivity. It depresses workers’ productivity or likelihood to become an entrepreneur over their whole life cycles.

5 Another potential channel not analyzed in this paper is automation. Acemoglu and Restrepo (2022) argue that aging that aggravates labor shortages may increase firms’ incentives to progressively develop and use modern technologies like robots that replace labor and increase its productivity.

6 See, among others, Glaeser et al. (1992), Jaffe et al. (1993), Audretsch and Feldman (1996), Carlino and Kerr (2015), Duranton and Puga (2020).

7 See, e.g., Liu and Westelius (2016). In Germany, productivity of the manufacturing sector was 77% higher than that of the household services sector (NACE 2 sectors O–T) in 2019, and productivity of the other services sectors (NACE 2 sectors G–N) was 54% higher.

8 Clustering at the county level yields similar results.

9 The data is from the “Arbeitskreis Volkswirtschaftliche Gesamtrechnungen der Länder” (November 2021 estimates), downloaded on Jan 10, 2023 from https://www.statistikportal.de/de/veroeffentlichungen/bruttoinlandsprodukt-bruttowertschoepfung-0.

10 Measuring aging by the change over time as in Daniele et al. (2019) rather than the level of average age may appear to be more intuitive. However, this allows capturing only the static effects of aging. The parameter of the change, ($\partial \hat{y}_{rt}/\partial \widehat{\mathrm{age}}_{rt}$), reflects only the effect on productivity growth of changes in the speed of aging but not that of aging itself. It is invariant to the actual age distribution. We rather hypothesize that older regions grow slower.

11 The data on the age composition of the population by 17 age cohorts is from the German Statistical office’s online database Genesis (https://www-genesis.destatis.de/genesis/online, Table 12411-0017), downloaded on Feb 8, 2022.

12 The data is from the “Arbeitskreis Volkswirtschaftliche Gesamtrechnungen der Länder” (November 2021 estimates), downloaded on Jan 10, 2023 from https://www.statistikportal.de/de/veroeffentlichungen/bruttoinlandsprodukt-bruttowertschoepfung-0.

13 Business services include sectors G–N of the NACE Rev. 2 classification (trade; transportation and storage; accommodation and food services; information and communication; finance and insurance; real estate; professional, scientific and technical services) while household services include sectors O–T (public administration, defense and social security; education; health and social services; arts, entertainment and recreation; other services).

14 We use the classification by the German Federal Institute for Research on Building, Urban Affairs and Spatial Development (BBSR, variable “Siedlungsstruktureller Regionstyp”, August 2018; file https://www.bbsr.bund.de/BBSR/DE/forschung/raumbeobachtung/Raumabgrenzungen/deutschland/kreise/siedlungsstrukturelle-kreistypen/download-ref-kreistypen-xls.xlsx, downloaded: Feb 5, 2022). Urban counties comprise all counties classified as “urban” region (“Städtische Region”) by the BBSR, nonurban counties comprise all other counties, including semi-urban regions (“Region mit Verstädterungsansätzen”) and rural regions (“Ländliche Region”).

15 Unfortunately, more disaggregated sectoral data that would allow us to better identify those services industries that benefit most from consumption by elderly people is not available for a sufficiently long period of time.

16 We use a row-standardized binary contiguity weights matrix (direct neighbors with a common border). The matrix was constructed from the SAS map dataset germany.sas7bdat, which is accessible only by licensed users of this software package.

17 More precisely, at an average age of 38 (46), which is the minimum (maximum) in our sample, a one-year increase is associated with a 0.12 (0.10) percentage points lower annual growth. These figures are calculated as $d\hat{y}_{rt}=\frac{\hat{\beta }}{\mathrm{age}_{rt}}d\mathrm{age}_{rt}$, where $d\mathrm{age}_{rt}=1$ is the assumed increase of average age.

18 As a consequence of this imprecision, the prob-value for the two being the same is only 0.103.

19 The point estimate for nonurban counties, which is not significant at conventional levels, suggests that a similar aging may be associated with a drop of annual productivity growth by only 0.10 percentage points. The variations within the two groups of counties are large, of course. The growth effects predicted by the changes in average age between 2000 and 2019 range from −0.87 to +0.38 percentage points among the urban counties, and from −0.54 to +0.12 percentage points among the nonurban counties.

20 This predicted effect is calculated as $100d\hat{y}_{rt}=100\frac{1}{{P65}_{rt}^{+}}\left[\hat{\gamma }_{P65+}+\hat{\gamma }_{\mathrm{sHH}{,}P65+}ln\left(\mathrm{sHH}_{rt}\right)\right]d{P65}_{rt}^{+}$, where ${P65}_{rt}^{+}$ denotes the initial-year share of the population aged 65+ (“share retired” in Fig. 1), $\hat{\gamma }_{P65+}=0.027$ its parameter, *sHH*_*r**t*_ the initial-year value-added share of household services, $\hat{\gamma }_{\mathrm{sHH}{,}P65+}=0.022$ the parameter of the interaction term, and $d{P65}_{rt}^{+}=0.1$ the assumed increase of the share of retirees (10 percentage points).

21 The correlation between average age of the workforce and the share of retirees is 0.65 (see Table 3).

22 See, among others, Humpert and Pfeifer (2013), Pfeifer and Wagner (2014).
